# Intraparietal esophageal leiomyomas diagnosed by endoscopic ultrasound-guided fine-needle aspiration cytology: Cytological and immunocytochemical features in two cases

**DOI:** 10.3892/ol.2014.2077

**Published:** 2014-04-16

**Authors:** P. TODARO, S.F. CRINÒ, A. IENI, S. PALLIO, P. CONSOLO, G. TUCCARI

**Affiliations:** 1 Department of Human Pathology ‘Gaetano Barresi’, University of Messina, Messina, I-98125 Italy; 2 Digestive Endoscopy Unit, Hospital Health Network ‘Polyclinic G. Martino’, University of Messina, Messina, I-98125 Italy

**Keywords:** esophageal leiomyomas, immunocytochemistry, cytopathology, cell block, differential diagnosis

## Abstract

Endoscopic ultrasound-guided fine-needle aspiration cytology (EUS-FNAC) has proven to be of significant value as a diagnostic method for the evaluation of esophageal mesenchymal tumors, such as true leiomyomas. Utilizing the cell block procedure, the present study reports the diagnostic approach of EUS-FNAC in two patients affected by this lesion, describing the cytological and immunocytochemical findings. Spindle-shaped elements with elongated nuclei were appreciable; moreover, the cytoplasmatic immunohistochemical positivity for smooth muscle actin and desmin strongly supported the diagnosis of leiomyoma when also taking into account the constant negativity for CD34, CD117 and S100. The differential diagnosis between spindle cell mesenchymal tumors and leiomyomas, and the clinico-therapeutic management of the latter are also discussed in the study.

## Introduction

Mesenchymal tumors of the gastrointestinal tract are uncommon, representing only a small percentage of gastrointestinal neoplasms (1–7). The tumors are generally localized within the submucosa as intramural nodules that can to lead to obstruction, ulceration and bleeding (3,8,9). The majority of these tumors, including leiomyomas, schwannomas and neurofibromas, show benign behavior, even if their malignant counterparts have been reported (2,7,8). Nevertheless, among spindle cell tumors, gastrointestinal stromal tumors (GISTs) represent the most commonly occurring event, but they are characterized by a different prognosis and clinical management, and therefore require a diagnostic distinction from the other entities, mainly from leiomyomas (3,7,10–14). The differential diagnosis between GISTs and gastrointestinal leiomyomas offers certain difficulties, not only due to their overlapping clinical and ultrasound presentations, but also due to their cytological appearance, largely represented by spindle cells.

Endoscopic ultrasound-guided fine-needle aspiration cytology (EUS-FNAC) has proven itself to be a reliable method for the diagnosis of GISTs (15) and other gastrointestinal mesenchymal tumors, including true leiomyomas (15). The present study reports two cases of true intramural leiomyomas of the esophagus, in which EUS-FNAC allowed the sampling of the submucosal lesions, which are otherwise difficult to biopsy by traditional methods; moreover, the immunophenotypic profile readily obtained from cell blocks aided in the definition of these lesions, distinguishing them from other gastrointestinal stromal or mesenchymal tumors. Patients provided written informed consent.

## Case reports

### Case 1

**Figure 1:**
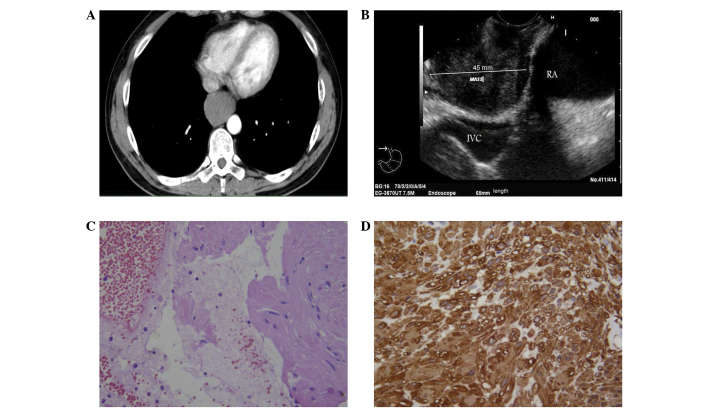
Case 1: (A) CT scan showing a hypodense mass with non-homogeneous contrast enhancement developing from the distal esophagus causing sub-stenosis of the lumen. (B) EUS scanning results revealing a 45-mm, hypoechoic round mass, originating from the muscle layer in contact with the inferior vena cava (IVC) and the right atrium (RA). (C) Cytological smear results exhibiting aggregates of spindle cell elements with elongated nuclei (hematoxylin and eosin staining; magnification, ×160). (D) The same elements were intensely immunoreactive for SMA (immunoperoxidase and Mayer’s hemalum counterstain; magnification, ×200). CT, computed tomography; EUS, endoscopic ultrasound; SMA, smooth muscle actin.

A 43-year-old male presented with dysphagia that had been apparent for 2 months. A physical examination revealed no abnormalities, and the standard serum laboratory tests were in the normal range. Computed tomography (CT) scans of the chest revealed a hypodense mass developing in the distal esophagus and causing substenosis of the lumen, which was extended to 65 mm in length, with non-homogeneous contrast enhancement (Fig. 1A). EUS examination revealed a 45-mm hypoechoic, round lesion with well demarcated margins, originating from the muscle layer of the distal esophagus in contact with the inferior caval vein and right atrium (Fig. 1B). EUS-FNAC was performed by using a convex array echoendoscope (EG-3870 UTK; Pentax, Co., Ltd., Tokyo, Japan) and by making two passes with a 22G needle. The specimens were processed by an in-room cytopathologist and immediately examined for adequate cellularity following staining by hematoxylin and eosin. A second slide was immediately fixed in 98% ethanol and stained with Papanicolaou. Any excess materials, including the needle and syringe utilized in the procedure, were rinsed in 10 ml 50% ethanol in a specimen container. All content was centrifuged in a 10-ml disposable centrifuge tube at 5,017 × g for 6 min to create 1 or 2 pellets; the supernatant fluid was decanted and the pelleted material was immediately fixed in a freshly prepared solution of 4% neutral buffered formalin for 45 min. The cell pellets were then placed in a cassette and stored at 80% ethanol until ready for processing in an automatic tissue processor (Leica TP1020; Leica, Buckinghamshire, UK). The cell blocks obtained were embedded in paraffin at 56°C, and 3-μm thick successive sections were cut and routinely stained by hematoxylin and eosin; parallel serial sections of the same thickness were mounted on silane-coated glasses and submitted to immunohistochemical procedures, as described previously (16,17).

### Case 2

**Figure 2:**
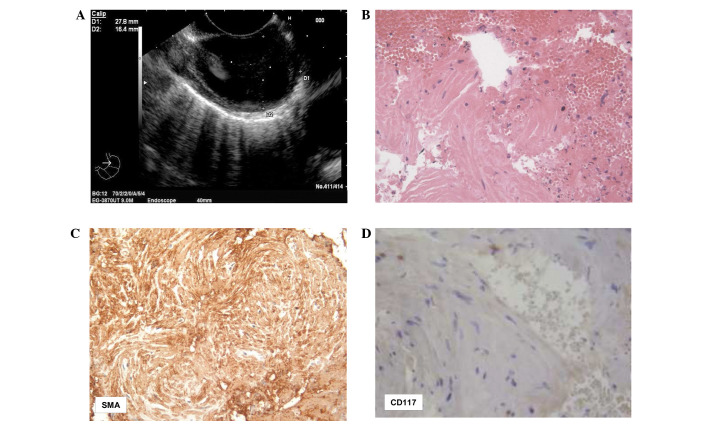
Case 2: (A) EUS scanning results revealing a 27.8×16.4-mm, hypoechoic, ovoid, well-delimited lesion originating from the muscle layer of the distal esophagus. (B) Clusters of spindle cells intermingled with red blood cells are indicative of leiomyoma (hematoxylin and eosin staining; magnification, ×160). (C) These elements were reactive for SMA (immunoperoxidase and Mayer’s hemalum counterstain; magnification, ×120), (D) while no immunoreactivity was found with CD117 (immunoperoxidase and Mayer’s hemalum counterstain; magnification, ×160). EUS, endoscopic ultrasound; SMA, smooth muscle actin.

A 39-year-old female presented with dyspepsia and esophageal reflux that had been apparent for 4 weeks. There was no weight loss, but nausea and mild vomiting were occasionally present. Upon physical examination, local peri-gastric discomfort and pain were noted. EUS scanning showed a 27.8×16.4-mm ovoid, homogeneous and hypoechoic well-delimited mass originating from the esophageal sub-mucosa (Fig. 2A). No lesions were evident elsewhere in the abdominal organs or lymph nodes. EUS-FNAC was performed with the same procedure as utilized in case 1; again, adequate cellularity and one cell block were obtained.

Following the FNAC procedures, the two patients were observed for a period of 48 h for any procedure-related complications.

### Cytological and immunocytochemical findings

The smears from the two cases exhibited a hemorrhagic background, with loose clusters or small aggregates of spindle-shaped cells (Figs. 1C and 2B) that had elongated nuclei, occasionally showing finely granular chromatin. No mitotic figures were found. The corresponding cell blocks documented an equivalent morphology characterized by small tissue fragments, with relatively low to moderate cellularity composed of monomorphic-uniform spindle cells, eosinophilic cytoplasm and vesicular nuclei (Figs. 1C and 2B). The nuclear chromatin was finely granular and evenly dispersed, while micronucleoli were inconspicuous. No atypia or mitoses were noted.

Immunohistochemical procedures were carried out on the 3-μm serial sections, utilizing the following commercially obtained antisera (all DakoCytomation, Copenhagen, Denmark): Vimentin [working dilution (w.d.), 1:250], smooth muscle actin (SMA; w.d. 1:200), desmin (w.d., 1:250), CD117 (w.d., 1:150), CD34 (w.d. 1:200), S-100 (w.d., 1:400) and Ki67 (MIB-1; w.d., 1:50). In each of the two cases, strong and diffuse cytoplasmic immunostaining was encountered for vimentin, desmin and SMA (Figs. 1D and 2C). No immunostaining was recorded for S100, CD34 and CD117 (Fig. 2D). The growth fraction, determined using Ki67 as the MIB-1 labeling index, was extremely low and quite inconspicuous, showing <1% positively-labeled nuclei.

In light of the microscopic examination and immunohistochemical findings, the two esophageal lesions were diagnosed as intraparietal true leyomiomas, without atypia. The patients refused surgical procedures, and were lost to follow-up subsequent to a period of 12 months.

## Discussion

It is well known that the diagnostic yield of EUS-FNAC greatly depends on the site, size and characteristics of the target tissues, as well as certain procedural aspects (9,15,18). By contrast, although conventional endoscopy and CT scans may identify esophageal lesions, these procedures cannot reveal the nature, size or origin of sub-mucosal neoplasms (7,9). However, the efficacy of EUS-FNAC as a main diagnostic procedure is also largely dependent on the expertise, training and interaction between the endosonographer and cytopathologist (15). In the present study, adequate cellular smears and corresponding cell blocks were obtained using the EUS-FNAC approach that is used on esophageal mesenchymal tumors, particularly true leiomyomas. Even if the observed spindle-shaped cells with elongated nuclei could also be confused with other gastrointestinal non-epithelial tumors, the serial immunohistochemical procedures performed on the cell blocks allowed acquisition of the final diagnosis. In fact, the coexistence of desmin and SMA strongly supported the smooth muscle nature of the observed esophageal neoplastic lesions, while the constant negativity for CD34, CD117 and S-100 excluded other diagnostic hypotheses, including inflammatory fibroid polyps, GISTs and schwannomas. Consequently, the availability of an adequate number of serial sections obtained from tissue blocks appears to be an additional diagnostic aid in order to perform the indicated immunohistochemical algorithm, as described previously (15,19,20). Finally, the low growth fraction, revealed by the Ki67 labeling index in the present study, further indicates the benign nature of leiomyomas, thus discounting the diagnostic hypotheses of highly malignant neoplasms, including leiomyosarcomas, spindle-cell amelanotic melanomas and undifferentiated sarcomatoid carcinomas (14,15).

Esophageal leiomyomas are rare benign tumors, with a frequent asymptomatic occurrence, that do not metastasize (21). In fact, patients with these tumors more commonly seek care due to difficulty in swallowing or as a result of the tumors being detected during the endoscopic workup for other diseases, as documented in case 2 of the present study. Moreover, the progression of these neoplasms shows a slow growing phase and the size of the lesions remains stable during the first year of follow-up. Therefore for those patients who refuse to receive surgical excision, as in the present cases, a periodic follow-up with EUS has been considered preferable and more accepted (22,23). On the other hand, the surgical treatment for esophageal leiomyomas depends on multiple factors, including tumor size, location, gross morphology and the patient’s symptoms and overall condition (21,24,25). Furthermore, indications for surgical treatment include unremitting symptoms, a progressive increase in tumor size, mucosal ulceration or the requirement to achieve the histopathological diagnosis due to an inconclusive EUS-FNAC procedure (25–27).

In summary, the present study provided further indications that EUS-FNAC has great clinico-diagnostic pre-surgical value, also allowing a correct differential diagnosis of other esophageal mesenchymal/stromal neoplasias with unpredictable biological behavior to be generated by immunohistochemistry.
